# Immune-checkpoint targeting drug conjugates: a novel class of promising therapeutic agents for cancer treatment

**DOI:** 10.1038/s41698-025-01011-7

**Published:** 2025-07-02

**Authors:** Silvia Marchesi, Arianna Marinello, Paolo Ambrosini, Chiara Cavalli, Giuseppe Lo Russo, Mario Occhipinti

**Affiliations:** 1 Department of Medical Oncology, Fondazione IRCCS Istituto Nazionale dei Tumori, Milan, Italy; 2 Department of Medical Oncology, International Center for Thoracic Cancers (CICT), Gustave Roussy, Villejuif, France; 3 INSERM Unit 1030 - Molecular Radiotherapy and Therapeutic Innovation, Gustave Roussy, Villejuif, France

**Keywords:** Cancer immunotherapy, Drug development, Cancer microenvironment

## Abstract

Immune-checkpoint targeting Drug Conjugates (IDCs) are a novel class of therapeutics that combine an immune checkpoint-targeting moiety, a cleavable linker, and a cytotoxic payload. By integrating features and functions of antibody-drug conjugates and immunotherapy, IDCs represent a promising strategy to remodel the tumor microenvironment and enhance antitumor efficacy. Several IDCs targeting checkpoints such as PD-L1, B7-H3, and B7-H4 are in early-phase clinical trials. This review summarizes available data on IDC efficacy and toxicity in human. Although current evidence is limited, ongoing phase III trials and biomarker studies will clarify their optimal clinical role, including potential for tumor-agnostic use.

## Introduction

Antibody-drug conjugates (ADCs) represent an advanced class of therapeutics designed to link cytotoxic agents to a targeted protein carrier, thereby enhancing specificity. Structurally, ADCs consist of three primary components: a monoclonal antibody (mAb), a linker, and a payload. The antibody is directed against a specific antigen, ideally one with restricted expression on tumor cell membranes^1^. Commonly used antibodies, such as IgG1, vary in affinity, size, and immunogenicity^2^. The linker serves as a bridge between the antibody and the payload, ensuring release within tumor cells while minimizing premature release into the plasma. Linkers are categorized as cleavable or non-cleavable based on their chemical properties^3^. Cytotoxic payloads, often tubulin inhibitors or DNA-damaging agents, are highly potent molecules. ADCs function through a combination of payload-dependent intracellular cytotoxicity and immune-mediated mechanisms, including complement-dependent cytotoxicity, antibody-dependent cytotoxicity, antibody-dependent cellular phagocytosis and bystander effect^4,5^. The drug-antibody ratio (DAR), defined as the average number of payloads linked to each antibody, is crucial for the efficacy and pharmacokinetics of ADCs. Specifically, low drug loading reduces the potency, while high payload loading can negatively affect pharmacokinetics and toxicity. Balancing DAR is therefore fundamental when constructing an active but tolerable ADC^6^.

Immunotherapy with immune checkpoint inhibitors (ICIs) as monotherapy or in combination with other agents has significantly improved the prognosis of various solid tumors and it is now used in both advanced and early-stage disease. Inhibitory immune receptors, commonly known as immune checkpoints, are essential for regulating immune responses. Key examples include Programmed Death Ligand 1 (PD-L1), Cytotoxic T-Lymphocyte Antigen 4 (CTLA-4), Lymphocyte-activation gene 3 (LAG3) and T-cell immunoreceptor with immunoglobulin and ITIM domain (TIGIT), with growing interest in emerging targets such as B7 Homolog 3 protein (B7-H3) and B7 Homolog 4 protein (B7-H4). As surface molecules, these checkpoints can be therapeutically targeted with blocking antibodies that prevent ligand-receptor interactions. By counteracting tumor immune escape mechanisms, these antibodies may help to reduce immune cell exhaustion or the accumulation of exhausted immune cells within the tumor microenvironment (TME), contributing to the restoration of anti-tumor immunity^7^. However, only patients with a “hot” TME benefit from ICIs, while the majority have a “cold” TME with lower immune cell recruitment and higher immunosuppressive cells and experience a poor response to ICIs. Therefore, huge efforts have been made to improve efficacy of ICIs, by combining them with other multiple therapeutic agents. However, this approach has not demonstrated great improvement in response rates, while increasing the risk of immune-related adverse events^8^.

**Fig. 1:**
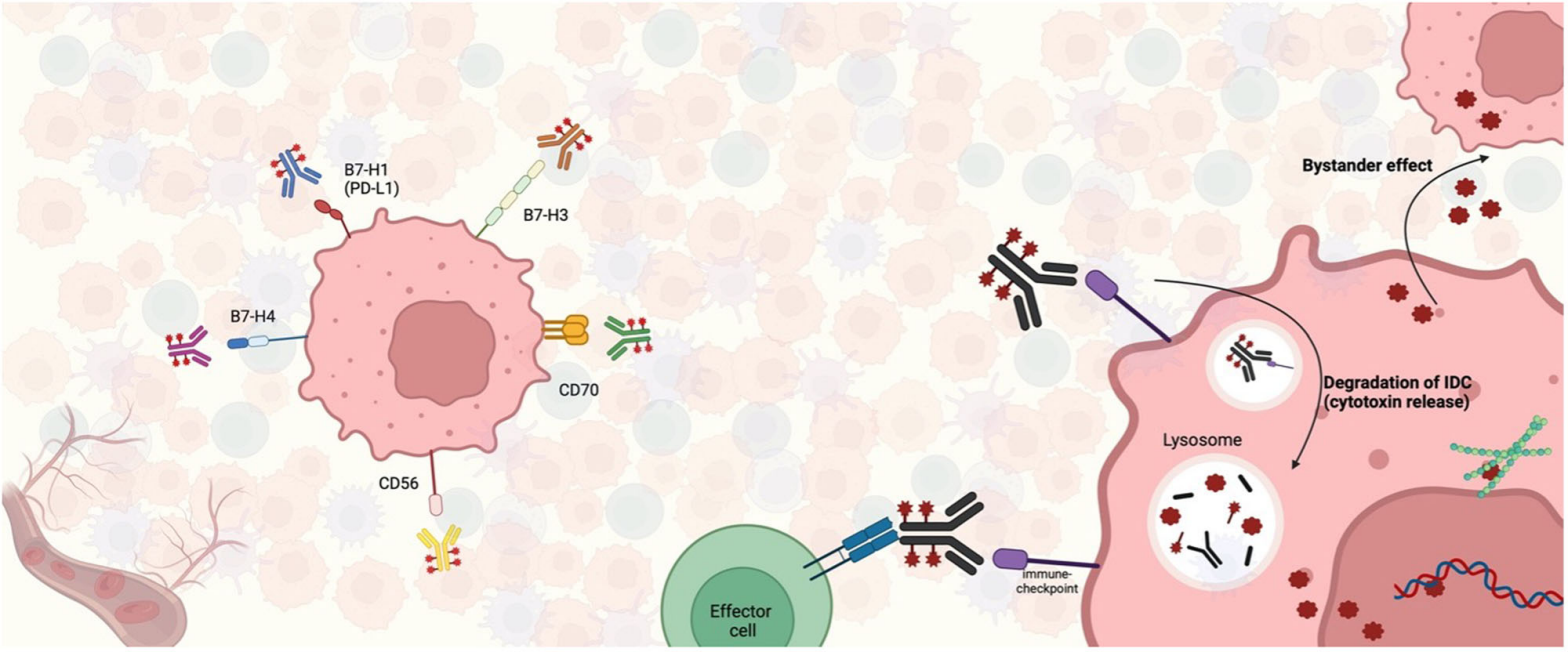
IDCs targets and mechanism of action within tumor microenvironment. IDCs recognize immune checkpoints such as PD-L1 (B7-H1), B7-H3, B7-H4, CD70, and CD56, expressed on the surface of tumor and tumor microenvironment cells. Upon binding to their target, IDCs are internalized and degraded within lysosomes, leading to the release of the cytotoxic payload inside the target cell. This results in direct tumor cell killing by microtubule disruption or DNA strand breakage, depending on the payload type. IDCs’ payload can also induce a bystander effect, impacting closer antigen-negative tumor cells. Additionally, IDCs may disrupt checkpoint interactions with effector immune cells, enhancing antitumor immune responses within tumor microenvironment.

Immune-checkpoint targeting Drug Conjugates (IDCs) are a novel class of promising anticancer therapeutic agents currently under evaluation in clinical trials. The tripartite complex structure of IDCs, consisting of an immune-checkpoint targeting moiety, a cleavable linker and a cytotoxic payload, which is similar to that of conventional ADCs, offers a promising strategy for remodeling the TME in cancer immunotherapy (Fig. 1 and Supplementary Fig. 1). Indeed, by combining the effects of ICIs which remove T-cell inhibitory signals and the release of the cytotoxic payload which induce multiple immunomodulatory mechanisms in TME, IDCs have the potential to enhance anticancer effects and thus improve patient outcomes.

**Fig. 2:**
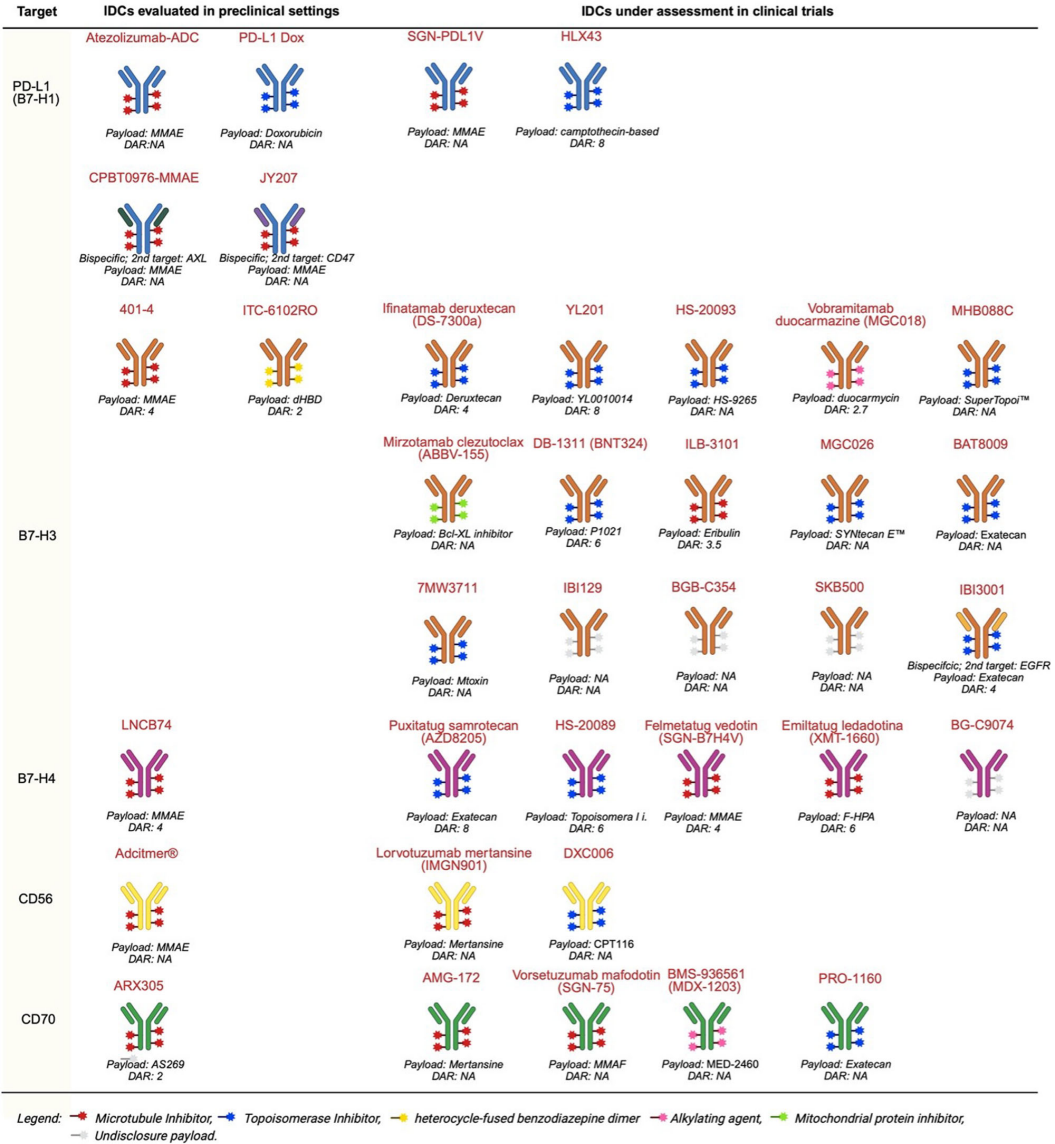
IDCs under investigation. MMAE monomethyl auristatin E, dHBD heterocycle-fused benzodiazepine dimer, F-HPA auristatin F-HPA, MMAF monomethyl auristatin phenylalanine, NA non available.

Among IDCs, those targeting PD-L1, B7-H3 and B7-H4 are currently the most extensively studied, with several compounds undergoing clinical investigation. Other immune checkpoints such as CD56, CD70 and CD73 are also being evaluated as potential targets for IDCs in solid tumors (Fig. 2). This review focuses on emerging IDCs, highlighting the biological rationale, summarizing the available clinical evidence, and exploring future research directions.

## PD-L1 (B7-H1)

PD-L1 (also known as CD274 and B7-H1), is a transmembrane glycoprotein that belongs to B7 family^9^. The PD-1/PD-L1 pathway is essential for establishing and maintaining immune tolerance within the TME. Binding of PD-1 to its ligands, PD-L1 and PD-L2, reduces T-cell activation, proliferation, and cytokine release, weakening the body’s anti-tumor immune response^10^. High PD-L1 expression is observed in various cancer types, including renal cell carcinoma (RCC), breast cancer, colorectal cancer (CRC), gastric cancer, non-small cell lung cancer (NSCLC), papillary thyroid cancer, and testicular cancer^11,12^. Several therapeutic antibodies targeting PD-L1 (e.g., atezolizumab, avelumab, durvalumab) and PD-1 (e.g., nivolumab, pembrolizumab, cemiplimab) are administered in clinical practice in various cancer types. As PD-L1 is widely expressed in several solid tumors, its targeting by anti-PD-L1 antibodies can facilitate the specific delivery of toxic payloads in various PD-L1-positive cancers [11]. Different IDCs targeting the PD-1/PD-L1 axis have been evaluated in preclinical studies, though only two have progressed to in-human clinical trials (Fig. 2).

SGN-PDL1V is a PD-L1-directed IDC composed of an anti-PD-L1 mAb conjugated to the microtubule-disrupting agent monomethyl auristatin E (MMAE) via a protease-cleavable peptide linker. SGN-PDL1V is currently being evaluated in SGNPDL1V-001 study (NCT05208762), a phase I clinical trial in pretreated patients with PD-L1-expressing solid tumors (Table 1). Fifty-five patients received SGN-PDL1V in the dose-escalation phase (0.5–1.75 mg/kg on days 1 and 8 of each 21-day cycle), including 54.5% with head and neck squamous cell carcinoma (HNSCC), 29.1% with NSCLC, 14.5% with triple-negative breast cancer (TNBC) and 1.8% with esophageal carcinoma. The overall incidence of grade 3 or higher ( ≥ 3 G3) toxicities was 30.9%, the most common of which was neutropenia, observed in 7.3% of cases. Treatment discontinuation due to treatment-emergent adverse events (TEAEs) occurred in 14.5% of patients. Confirmed objective response rate (ORR) assessed across all doses and tumor types was 12.7%, with a median duration of response (DOR) of 7.9 months. Objective responses were independent of PD-L1 expression levels. ORR were 33.3% in NSCLC with 1.5 mg/kg dose and in 42.9% in HNSCC with the 1.75 mg/kg dose. 1.5 mg/kg was identified as the recommended dose for expansion (Table 1). Given these promising preliminary findings regarding antitumor activity, enrollment in the phase I trial is ongoing^13^.

**Table 1:** Preliminary results of clinical trials evaluating IDCs

TARGET	DRUG NAME	TRIAL NAME	TRIAL PHASE	REGIMEN	TUMOR TYPE	PATIENTS	RESULTS	Adverse Events ≥ G3
PD-L1 (B7-H1)	SGN-PDL1V	SGNPDL1V-001 (NCT05208762)^13^	I	Monotherapy (dose escalation 0.5–1.75 mg/kg d1,8 q3W)	HNSCC, NSCLC, TNBC, esophageal carcinoma	*N* = 55 Pretreated	ORR : 27.3% in all tumors (12.7% confirmed); 33.3% (1.5 mg/kg) in NSCLC; 42.9% (1.75 mg/kg) in HNSCC	TRAEs: 23.4% Decreased neutrophil count: 7.3%
B7-H3 (CD276)	Ifinatamab deruxtecan (DS-7300a)	IDeate-Pantumor01 (NCT04145622)^17^	I/II	Monotherapy (dose escalation: 0.8–16.0 mg/kg q3W),	HNSCC, ESCC, squamous NSCLC, SCLC, bladder cancer, sarcomas, endometrial cancer, melanoma, mCRPC, breast cancer	*N* = 174 Pretreated	ORR: - ESCC: 21.4% - mCRPC: 25.4% - SCLC: 52% - sqNSCLC: 30.8%	TEAEs: 43.7%; Anemia: 19.0%; Neutropenia: 4.0%; Nausea: 3.4%; Lymphocyte count decreased: 3.4%
	Ifinatamab deruxtecan (DS-7300a)	IDeate-Lung01 (NCT05280470)^18^	II, randomized	Monotherapy (8 mg/kg q3W vs 12 mg/kg q3W)	SCLC	*N* = 88 Pretreated	ORR: - 8 mg/kg cohort: 26.1% - 12 mg/kg cohort: 52.4%	TEAEs: 39.1% (8 mg/kg cohort) vs 45.2% (12 mg/kg cohort)
	YL201	NCT06057922 and NCT05434234^20^	I/II	Monotherapy (dose escalation: 0.8–3.0 mg/kg q3W)	Solid tumors	*N* = 312 Pretreated	ORR: - Extensive stage SCLC: 63.9% - NPC: 48.6% - NSCLC (Adenocarcinoma): 28.6% - NSCLC (SCC): 8.3% NSCLC (LELC): 54.2%	TRAE: 54.5% Leukopenia: 29.5%, Anemia: 25.0%, Neutropenia: 31.7%, Thrombocytopenia:13.8%.
	HS-20093	ARTEMIS-001 (NCT05276609)^22^	I	Monotherapy (dose escalation: 1–16 mg/kg q3W)	Solid tumors	*N* = 53 Pretreated	ORR: 30.0%	neutrophil count decreased, white blood cell count decreased, lymphocyte count decreased, platelet count decreased
	HS-20093	ARTEMIS-002 (NCT05830123)^24^	II, randomized	Monotherapy (8 mg/kg vs 12 mg/kg q3W)	Sarcomas	*N* = 21 Pretreated	ORR (12.0 mg/kg): 20.0%	Neutropenia, leukopenia, thrombocytopenia, anemia
	Vobramitamab duocarmazine (MGC018)	NCT03729596^26^	I/II	Monotherapy (0.5–4 mg/kg q3W) or in combination with MGA012	Solid tumors	*N* = 32 Pretreated	ORR (3 mg/kg): - mCRPC: 25% - NSCLC: 25%	TRAE: 50% Neutropenia: 22.1%, Thrombocytopenia: 7%, Anemia: 5.8%
	Vobramitamab duocarmazine (MGC018)	TAMARACK (NCT05551117)^27^	II, randomized	Monotherapy (2.9 mg/kg vs 2.7 mg/kg q4W)	mCRPC and other solid tumors	*N* = 181 Pretreated	ORR (2.0 mg/kg): 20.0% ORR (2.7 mg/kg): 40.6%	TRAEs: 46.7% (2.0 mg/kg) vs 52.3% (2.7 mg/kg)
	Mirzotamab clezutoclax (ABBV-155)	NCT03595059^28^	I	Monotherapy or in combination with taxanes	Solid tumors	Monotherapy *N* = 45 Combination *N* = 92 Pretreated	ORR: - Monotherapy: 0% - Combination: 14%	Anemia: 3% Lymphocyte count decreased: 3% Fatigue: 3% Diarrhea: 3%
	MHB088C	CTR20231298^30^	I/II	Monotherapy (0.8, 1.6, 3.0 mg/kg q2W; 3.0, 4.0 mg/kg q3W)	Solid tumors	*N* = 60 Pretreated	ORR: 41.7%	Neutrophil count decreased: 33.3%, White blood cell count decreased: 26.7%, platelet count decreased: 23.3% Anemia: 15.0%
	DB-1311/ BNT324	NCT05914116^31^	I/II	Monotherapy (dose escalation 3–12 mg/kg q3W)	Solid tumors	*N* = 277 Pretreated patients	ORR: 32.4% DCR: 82.4%	TRAE: 91.7% Neutrophil count decreased: 20.2% Anemia: 7.1% Platelet count decreased: 6.3%
B7-H4	Puxitatug samrotecan (AZD8205)	BLUESTAR (NCT05123482)^34^	I/II	Monotherapy (dose escalation :0.8–3.2 mg/kg q3W)	Biliary tract cancer, breast cancer, ovarian cancer, endometrial cancer	*N* = 47 Pretreated	ORR: 19.5%	TRAE: 55.3% Neutropenia: 34.0% Thrombocytopenia: 7.6% Anemia: 4.3%
	HS-20089	NCT05263479^35^	I	Monotherapy (dose escalation: 0.7–7.2 mg/kg q3W)	Solid tumors	*N* = 52 Pretreated	ORR: - TNBC: 28.6%	N/A
	Felmetatug vedotin (SGN-B7H4V)	SGNB7H4V-001 (NCT05194072)^36^	I	Monotherapy (0.75, 1.0, 1.25, or 1.5 mg/kg d1,8 q3W or 1.25, 1.5, 1.75, or 2.0 mg/kg d1,15 q4W).	Solid tumors	*N* = 75 Pretreated	Confirmed objective responses - Breast: 7/25 - Ovarian: 2/15 - Endometrial: 1/16 - Biliary tract: 2/9	In 2Q3W Neutropenia: 14.3% In 2Q4W Anemia: 5.0% Dyspnea: 5.0% Hypotension: 5.0% Pneumonia 5.0%
CD56	Lorvotuzumab mertansine (IMGN901)	NCT00065429^39^	I	Monotherapy (dose escalation 4-94 mg/mq d1-3 q3W)	SCLC, neuroendocrine lung tumors, MCC, carcinoid tumors, and other CD56+ tumors	*N* = 97 Pretreated	PFS: 2.1 mo.	TRAEs 80.4% Hyponatremia (8.2%), Dyspnea (8.2%), Elevated GGT (7.2%)
	Lorvotuzumab mertansine (IMGN901)	NCT01237678^40^	I/II	Lorvotuzumab mertansineLM+carboplatin-etoposide (arm 1) Carboplatin-etoposide (arm 2)	SCLC	*N* = 94 (arm1) 47 (arm 2) Pretreated	PFS (HR 0.93) - Arm 1: 6.2 months - Arm 2: 6.7 months OS: - Arm 1: 10.1 months - Arm 2: 11.0 months ORR: Arm 1: 67% Arm 2: 59%	TRAEs 88% (arm 1) 70% (arm 2) Neutropenia (48.9% arm 1, 44.7% arm 2); Anemia (19.1% arm 1, 21.3% arm 2); Peripheral Sensory Neutropathy (18.1% arm 1; 0% arm 2) Thromocytopenia (17% arm 1, 21.2% arm 2)
CD70	AMG-172	NCT01497821^42^	I	Monotherapy (dose escalation: 0.15–2.4 mg/kg q2W)	ccRCC	*N* = 37 Pretreated	ORR: 5.4%	N/A
	Vorsetuzumab mafodotin (SGN-75)	NCT01015911^43^	I	Monotherapy (dose escalation0.3–4.5 mg/kg q3W or 0.3–0.6 mg/kg weekly)	ccRCC and B-Cell Non-Hodgkin’s Lymphoma	*N* = 58	ORR: - NHL: 7% - ccRCC: 6%	AE with q3W dosage: 70% AE with weekly dosage: 55%
	BMS-936561 (MDX-1203)	NCT00944905^44^	I	Monotherapy (dose escalation: 0.5-15 mg/kg q3W)	ccRCC and B-Cell Non-Hodgkin’s Lymphoma	*N* = 26	ORR: 0%	TRAEs: 34.6%

*HNSCC* head and neck squamous cell carcinoma, *NSCLC* non-small cell lung cancer, *TNBC* triple negative breast cancer, *ORR* objective response rate, *TRAEs* treatment related adverse events, *ESCC* esophageal squamous-cell carcinoma, *SCLC* small cell lung cancer, *mCRPC* metastatic castration resistant prostate cancer, *TEAEs* treatment emergent adverse events, *SCC* squamous cell carcinoma, *LELC* lymphoepithelioma-like carcinoma, *DCR* disease control rate, *MCC* Merkel cell carcinoma, *PFS* progression free survival, *OS* overall survival, *ccRCC* clear cell renal cell carcinoma.

## B7-H3

B7-H3, also known as CD276 and B7RP-2, is a type 1 transmembrane glycoprotein whose expression is limited in normal tissues but increased in a wide range of cancers^14^. B7-H3 promotes cancer cell proliferation, cell migration and invasion and cell differentiation. Pathologic angiogenesis via B7-H3 signaling has also been described, yielding the basis for combination treatment with anti-B7-H3 drugs and antiangiogenic therapies^15^. Furthermore, B7-H3 plays an important role in adaptive immunity inhibition by reducing natural killer (NK) cell activation. Additionally, when overexpressed on antigen presenting cells, B7-H3 reduces CD4 and CD8 T-cell activation and effector cytokine release but enhances immunosuppressive TME^16^. Several IDCs targeting B7-H3 are under evaluation in clinical trials (Tables 1, 2), while others have only been tested in preclinical settings (Fig. 2).

**Table 2:** Ongoing clinical trials evaluating IDCs

TARGET	DRUG NAME	TRIAL NAME	TRIAL PHASE	REGIMEN	TUMOR TYPE
PD-L1 (B7-H1)	HLX43	NCT06115642	I	Monotherapy Pretreated patients	Solid tumors
		NCT06848699	Ib/II	In combination with Serplulimab Pretreated patients	Solid tumors
		NCT06839066	II	Monotherapy Pretreated patients	NPC
		NCT06857279	II	Monotherapy Pretreated patients	HNSCC
		NCT06769152	II	Monotherapy Pretreated patients	Cervical cancer
		NCT06769113	II	Monotherapy Pretreated patients	ESCC
		NCT06742892	II	Monotherapy Pretreated patients	HCC
B7-H3 (CD276)	Ifinatamab deruxtecan (DS-7300a)	IDeate-PanTumor02 (NCT06330064)	Ib/II	Monotherapy, Pretreated patients	Endometrial cancer; HNSCC; pancreatic ductal adenocarcinoma; CRC; HCC; adenocarcinoma of esophagus, gastroesophageal junction, and stomach; urothelial carcinoma; ovarian cancer; cervical cancer; biliary tract cancer; HER2-low breast cancer; cutaneous melanoma.
		IDeate-Lung02 (NCT06203210)	III	Monotherapy vs standard treatment of physician’s choice, Pretreated patients	SCLC
		IDeate-Lung03 (NCT06362252)	Ib/II	In combination with atezolizumab ± carboplatin, First-line induction or maintenance	SCLC
		IDeate-Esophageal01 (NCT06644781)	III	Monotherapy vs investigator’s choice of chemotherapy, Pretreated patients	ESCC
		KEYMAKER-U01 Substudy 01 A (NCT04165070)	I/II	In combination with pembrolizumab ± carboplatin (part B), Treatment naïve patients	NSCLC
		NCT04471727	I/II	In combination with MK-6070, Pretreated patients	SCLC
		IDeate-Prostate02 (NCT06863272)	I/II	Monotherapy or in combination with MK-5684 or Abiraterone/Enzalutamide, Pretreated patients	mCRPC
		NCT06780137	Ib/II	Monotherapy or in combination with Gacatamig (MK-6070), Pretreated patients	SCLC
	YL201	NCT06241846	II	Monotherapy Pretreated or non-pretreated patients	mCRPC
		NCT06394414	I	In combination with serplulimab with or without platinum-based chemotherapy, Pretreated or non-pretreated patients	Solid tumors
		NCT06612151	III	Monotherapy vs topotecan hydrochloride Pretreated patients	SCLC
		NCT06629597	III	Monotherapy vs investigator’s choice of chemotherapy, Pretreated patients	NPC
	HS-20093	ARTEMIS-003 (NCT06001255)	II	Monotherapy Pretreated patients	mCRPC and other solid tumors
		ARTEMIS-005 (NCT06112704)	II	Monotherapy Pretreated patients	Esophageal carcinoma or other advanced solid tumors
		ARTEMIS-006 (NCT06007729)	II	Monotherapy Pretreated patients	HNSCC and other solid tumors
		ARTEMIS-007 (NCT06052423)	II (Withdrawn due to research and development strategy adjustment)	Monotherapy Non-pretreated patients	SCLC
		ARTEMIS-008 (NCT06498479)	III	Monotherapy vs topotecan Pretreated patients	SCLC
		ARTEMIS-009 (NCT06526624)	III	Monotherapy as consolidation therapy vs active surveillance	Limited stage SCLC
		ARTEMIS-101 (NCT06332170)	I	In combination with Adebrelimab or cetuximab or Enzalutamide ± Platinum-containing chemotherapy Pretreated or non-pretreated patients	Solid tumors
		ARTEMIS-103 (NCT06699576)	Ib	In combination with adebrelimab or anlotinib and/or epirubicin, Pretreated patients	Bone and Soft Tissue Sarcoma
		ARTEMIS-102 (NCT06825624)	Ib	In combination with bevacizumab and capecitabine or 5-fluorouracil or 5-fluorouracil and oxaliplatin, Pretreated or non-pretreated patients	CRC
		NCT06621563	Ib	In combination with HS-20117 ± Platinum-containing chemotherapy Pretreated patients	Solid tumors
		NCT06551142	I	Monotherapy or in combination with platinum or atezolizumab or pembrolizumab or durvalumab or cetuximab or bevacizumab Pretreated patients	Solid tumors
		NCT06885034	Ib/II	Monotherapy Pretreated patients	CRC
		NCT05277051	I	Monotherapy or in combination with dostarlimab and/or belrestotug ± nelistotug Pretreated patients	HNSCC, NSCLC, RCC, breast cancer, gastric cancer, CRC, endometrial cancer, ovarian cancer
	Vobramitamab duocarmazine (MGC018)	NCT05293496	I	In combination with MGD019/lorigerlimab Pretreated patients	Solid tumors
		NCT06227546	II	Monotherapy Pretreated patients	SCLC
	MHB088C	NCT05652855	I/II	Monotherapy Pretreated patients	Solid tumors
	ILB-3101	NCT06426680	I/II	Monotherapy Pretreated patients	Solid tumors
	MGC026	NCT06242470	I/II	Monotherapy Pretreated patients	Solid tumors
	IBI3001	NCT06349408	I/II	Monotherapy Pretreated patients	Solid tumors
	BAT8009	NCT05405621	I	Monotherapy Pretreated patients	Solid tumors
	7MW3711	NCT06008379	I/II	Monotherapy Pretreated patients	Solid tumors
		NCT06008366	I/II	Monotherapy Pretreated patients	Solid tumors
	IBI129	NCT05991349	I/II	Monotherapy Pretreated patients	Solid tumors
	BGB-C354	NCT06422520	I	Monotherapy or in combination with tislelizumab Pretreated patients	Solid tumors
	SKB500	NCT06736327	I	Monotherapy Pretreated patients	Solid tumors
B7-H4	HS-20089	NCT06014190	II	Monotherapy Pretreated patients	Ovarian and endometrial cancer
		NCT06336707	I	In combination with adebrelimab ± platinum or bevacizumab ± platinum Pretreated patients	Solid tumors
		NCT06855069	III	Monotherapy vs investigator’s choice of chemotherapy, Pretreated patients	Platinum-resistant Recurrent Epithelial Ovarian Cancer, Fallopian Tube Cancer, or Primary Peritoneal Cancer
	Emiltatug Ledadotin (XMT-1660)	NCT05377996	I	Monotherapy Pretreated patients	Breast, endometrial, and ovarian cancer
	BG-C9074	NCT06233942	I	Monotherapy and in combination with tislelizumab Pretreated patients	Solid tumors
	LNCB74	NCT06774963	I	Monotherapy Pretreated patients	Solid tumors
CD56	DXC006	NCT06224855	I	Monotherapy Pretreated patients	Solid and hematologic tumors
CD70	PRO-1160	NCT05721222	I	Monotherapy Pretreated patients	RCC, NPC, non-Hodgkin lymphoma
CD73	BB-1709	NCT06241898	I	Monotherapy Pretreated patients	Solid tumors

*HNSCC* head and neck squamous cell carcinoma, *CRC* colorectal cancer, *HCC* hepatocellular carcinoma, *ESCC* esophageal squamous-cell carcinoma, *SCLC* small cell lung cancer, *NSCLC* non-small cell lung cancer, *mCRPC* metastatic castration resistant prostate cancer, *RCC* renal cell carcinoma, *NPC* nasopharyngeal carcinoma.

Ifinatamab deruxtecan (I-DXd), previously known as DS-7300a, is composed of a humanized mAb directed against B7-H3, conjugated via an enzymatically cleavable tetrapeptide-based linker to deruxtecan, a cytotoxic DNA topoisomerase I inhibitor, with an average DAR of 4. The IDeate-Pantumor01 (NCT04145622) trial is an ongoing phase I/II study enrolling patients with advanced or unresectable solid tumors refractory to prior treatments (Table 1) to evaluate I-DXd administered every three weeks from a starting dose of 0.8 mg/kg. Preliminary data from 174 patients (97 in the dose-escalation cohort and 77 in the dose-expansion cohort) receiving I-DXd showed an acceptable safety profile with TEAEs ≥ G3 reported in 43.7% of patients. Drug discontinuation due to TEAEs occurred in 8.0% of the cases, interruption in 21.8% and dose reduction in 10.3%. One patient with endometrial cancer receiving I-DXd at 16.0 mg/kg experienced a grade 5 (G5) interstitial lung disease (ILD). The most common ≥G3 TEAEs were anemia (19.0%), neutropenia (4.0%), and nausea (3.4%). The dose selected for the dose-expansion cohort was 12.0 mg/kg administered every three weeks. ORRs ranged from 21.4 to 30.8% in esophageal squamous-cell carcinoma, metastatic castration resistant prostate cancer (mCRPC), and NSCLC, whereas they reached 52.0% in small cell lung cancer (SCLC). B7-H3 expression level was available in 17 patients with SCLC and 62 patients with mCRPC, with moderate to high levels in all participants. No trend of association between best overall tumor response and B7-H3 intensity was found in either SCLC or mCRPC cohorts^17^. The promising data reported in the SCLC cohort led to the initiation of several other trials in this tumor type. The IDeate-Lung01 (NCT05280470) is an ongoing phase II trial that randomizes pretreated patients with extensive-stage (ES) SCLC to receive I-DXd at 8 mg/kg or 12 mg/kg doses administered every three weeks (Table 1). An interim analysis evaluating 88 patients showed that the incidence of any-grade and ≥ G3 TEAEs was slightly higher in the 12 mg/kg group than in the 8 mg/kg group (97.6% vs 93.6% and 45.2% vs 39.1%, respectively). The most common TEAEs were gastrointestinal, hematological toxicities and fatigue. Incidence of ILD was similar between the two cohorts, occurring in 8.7% of cases with the 8 mg/kg dose and 7.1% with the 12 mg/kg dose^18^. A higher ORR was reported in the 12 mg/kg group than in the 8 mg/kg group (52.4% vs 26.1%), while the disease control rate (DCR) was similar in both groups (80.4% in the 8 mg/kg group and 90.5% in the 12 mg/kg group), as well as median progression-free survival (mPFS) (4.2 vs 5.5 months) and overall survival (OS) (9.4 vs 11.8 months). Moreover, a subset analysis of 37 patients with brain metastases demonstrated promising intracranial efficacy. The central nervous system (CNS) ORR in 21 patients who did not receive local radiotherapy was 33% with I-DXd 8 mg/kg and 50% with 12 mg/kg^19^. Table 2 summarizes other currently ongoing trials evaluating the efficacy and safety of I-DXd in patients with solid tumors as monotherapy or in combination with other drugs.

YL201 is an anti-B7-H3 IDC composed of a humanized mAb conjugated to a novel topoisomerase 1 inhibitor (YL0010014) via a protease cleavable linker, with a DAR of 8. Two ongoing phase I clinical trials (NCT06057922, NCT05434234) are evaluating YL201 at different doses (0.8–3.0 mg/kg every three weeks) in pretreated patients with advanced solid tumors, including ES-SCLC, nasopharyngeal cancer (NPC), NSCLC, and esophageal carcinoma (Table 1). 312 patients received at least one dose; treatment related adverse events (TRAEs) of any grade and ≥ G3 were reported in 97.1% and 54.5% of the cases, respectively. TRAEs led to YL201 dose reduction in 17.0% and drug discontinuation in 5.4%. TRAEs were mainly hematological, including ≥G3 leukopenia in 29.5%, ≥ G3 anemia in 25.0%, ≥ G3 neutropenia in 31.7% and ≥G3 thrombocytopenia in 13.8%. Treatment-related ILD was observed in only four patients (1.3%). Among the 287 patients with post-baseline tumor assessments, the ORR was 40.8%, while the DCR was 83.6%, with a mPFS of 5.9 months. In the ES-SCLC cohort, YL201 demonstrated encouraging clinical activity, achieving an ORR of 63.9% and a DCR of 91.7% with a mPFS of 6.3 months. In NPCs, ORR and DCR were 48.6% and 92.9%, with a mPFS of 7.8 months. Elevated rate of ORR was also reported in primary lung lymphoepithelioma-like carcinoma (54.2%), while ORR were lower in lung adenocarcinoma (28.6%) and in squamous cell lung cancer (8.3%)^20^. Notably, intracranial response was reported in 30.0% (3/10) of patients with ES-SCLC and brain metastases and in 33.3% (1/3) of patients with NSCLC and CNS metastases, highlighting the potential efficacy of YL201 in this particularly challenging subgroup^21^. Expression levels of B7-H3 on tissue samples and concentration of soluble B7-H3 in blood were evaluable in 152 and 223 patients, respectively. No significant correlation with clinical response was demonstrated for both markers^20^. Several trials assessing safety and efficacy of YL201 alone or in combination with other drugs are currently ongoing as shown in Table 2.

HS-20093 is composed of a human immunoglobulin G1 mAb directed against B7, linked to a cytotoxic agent. The ARTEMIS-001 (NCT05276609) is a first-in-human phase I trial of HS-20093 in patients with advanced solid tumors who have received prior standard of care treatments (Table 1). Fifty-three patients were enrolled in the dose escalation planned cohorts (1.0–16.0 mg/kg administered every three weeks) and the maximum tolerated dose (MTD) was determined to be 12.0 mg/kg. TEAEs were reported in all patients, the most common of which were neutropenia and thrombocytopenia while no cases of ILD were reported. Among the 50 patients with evaluable tumor responses, the ORR was 30.0% and the DCR was 86.0%, with a mPFS of 5.4 months^22^. Considering the 56 patients with extensive stage SCLC enrolled in both the dose escalation and the expansion arms of ARTEMIS-001 trial and treated with doses at 8.0 mg/kg (*n* = 31) or 10.0 mg/kg (*n* = 25) of HS-20093, the ORR was 61.3% for patients at the 8.0 mg/kg dose and 50.0% for patients at the 10.0 mg/kg dose. DCR and mPFS at 8.0 and 10.0 mg/kg dose were 80.6% and 95.5%, 5.9 and 7.3 months, respectively. OS was 9.8 months in patients receiving 8.0 mg/kg while it was not reached in patients at the 10.0 mg/kg dose. B7-H3 expression levels did not correlate with objective tumor response; however, patients with high B7-H3 immunohistochemistry (IHC) expression ( ≥ 1%) showed a trend towards a longer mPFS^23^. ARTEMIS-002 (NCT05830123) is a phase II trial evaluating HS-20093 in patients with relapsed or refractory osteosarcoma and other sarcomas, randomized to receive either 8 mg/kg (*n* = 15) or 12 mg/kg (*n* = 19) (Table 1). The safety profile was in line with that previously reported, with hematological adverse events being the most common ≥ G3 toxicity. Among the 21 patients with evaluable responses, the ORR and the DCR at 12.0 mg/kg were 20.0% and 100% respectively, while the DCR at 8 mg/kg was 81.8%. There was no correlation between the level of B7-H3 expression and tumor response^24^. HS-20093 alone or in combination with other compounds is under evaluation in many other studies, including two phase III trials, as shown in Table 2.

Vobramitamab duocarmazine, also known as MGC018, is a novel IDC composed of anti-B7-H3 human mAb conjugated to the cleavable linker duocarmycin payload duocarmazine. In humans, vobramitamab duocarmazine was firstly evaluated in a phase I/II trial (NCT03729596) administered alone or in combination with MGA012, an anti-PD-1 antibody, in patients with advanced solid tumors (Table 1). Results of the dose-escalation MGC018 monotherapy part of the 6 planned dose cohorts (0.5 mg/kg – 4 mg/kg administered every three weeks), reported TEAEs in all 29 patients enrolled, the most common of which were anemia, neutropenia, fatigue and hyperpigmentation. The recommended phase II dose was determined to be 3 mg/kg administered on days 1 and 22 of cycle 1 and every subsequent 42-day cycle thereafter^25^. Among the 86 patients who had received at least one dose in the expansion cohort, 90.7% experienced at least one TRAE and 50.0% a TRAE ≥ G3. TRAEs led to discontinuation in 7.0%, dose reduction in 20.9% and dose interruption in 45.3%. The most frequent ≥ G3 TRAE was neutropenia (22.1%), followed by thrombocytopenia (7.0%) and anemia (5.8%). Thirty-two of the patients enrolled in the expansion cohort were evaluable for tumor response (mCRPC, *n* = 16 and NSCLC, *n* = 16), with an ORR of 25% in both the mCRPC and NSCLC cohorts^26^. The phase II TAMARACK trial (NCT05551117) randomized mCRPC to receive vobramitamab duocarmazine at a dose of 2.0 mg/kg or 2.7 mg/kg every four weeks (Table 1). 181 patients received the treatment; ≥ G3 TRAEs occurred in 46.7% of patients at the dose of 2.0 mg/kg and 52.3% at the dose of 2.7 mg/kg. G5 AEs were pneumonitis (*n* = 3), heart failure, stress cardiomyopathy, ventricular fibrillation, pleural effusion, and gastrointestinal hemorrhage (*n* = 1 each). ORR was 20.0% in patients treated in the 2.0 mg/kg arm and 40.6% in the 2.7 mg/kg arm^27^. Two other trials evaluating Vobramitamab duocarmazine in different settings are ongoing, although results have not been published yet (Table 2).

Mirzotamab clezutoclax (Mirzo-C), also known as ABBV-155, is composed of anti-B7-H3 mAb conjugated, via a solubilizing linker, to a B-cell lymphoma extra-long (Bcl-XL) inhibitor. Mirzo-C was investigated in a phase I trial (NCT03595059) alone or in combination with taxane therapy in patients with relapsed and/or refractory solid tumors (Table 1). In the dose escalation cohort, 31 patients received Mirzo-C alone, while 28 patients received the combination therapy. No dose-limiting toxicities were documented with monotherapy, while two patients receiving the combination therapy developed a G4 neutropenia related to paclitaxel. ≥ G3 AEs overall included anemia, decreased lymphocyte count, fatigue, and diarrhea (3% each). ORR was 0% in monotherapy arm and 14% with combination, while DCR was of 52% and 68%, respectively^28^. In the dose-expansion phase, patients with SCLC were treated with Mirzo-C monotherapy (*n* = 14), NSCLC with Mirzo-C + docetaxel (*n* = 36), and hormone-positive, HER-2-negative, post–CDK4/6 inhibitor breast cancer with Mirzo-C + paclitaxel (*n* = 28). Neutropenia was not observed in the SCLC monotherapy cohort but was common with paclitaxel combination in breast cancer ( ≥ G3 29.0%) and docetaxel combination in NSCLC ( ≥ G3 44.0%). The ORR was 0% in SCLC, 11% in NSCLC and 18% in breast cancer, while the DCR was 7% in SCLC; 81% in NSCLC, and 71% in breast cancer^29^.

MHB088C is an anti-B7-H3 mAb conjugated via a cleavable linker with an undisclosed topoisomerase-1 inhibitor. The phase I/II CTR20231298 study enrolled Asian patients with recurrent or metastatic solid tumors (Table 1). 60 patients received at least one dose of MHB088C, 14 in the dose escalation cohort (0.8–3.0 mg/kg every two weeks; 3.0-4.0 mg/kg every three weeks) and 46 in the dose expansion cohort (3.0 mg/kg administered every three weeks). The most common ≥ G3 TRAEs were decreased neutrophil count (33.3%), thrombocytopenia (23.3%) and anemia (15.0%), while no cases of ILD were reported. Among the 12 patients with an evaluable response, the ORR was 41.7% and the DCR was 91.7%^30^. A parallel trial is underway to assess MHB088C in Caucasian patients (Table 2).

DB-1311/BNT324 is a novel topoisomerase-I-inhibitor-based IDC targeting B7-H3. A dose escalation phase I/II clinical trial is ongoing (NCT05914116) to evaluate MTD and safety profile. Among the 277 pretreated patients with solid tumors which received at least one dose of DB-1311/BNT324 across 5 dose cohorts (between 3 and 12 mg/kg every three weeks), MTD was established at 9 mg/kg. TRAEs occurred in 91.7% of the patients, with ≥ G3 TRAEs reported in 41.5% of the patients. In 9.0% of the patient TRAEs led to dose reduction, 15.9% to treatment interruption and in 5.4% to treatment discontinuation. One patient receiving DB-1311/BNT324 at a dose of 9 mg/kg died due to treatment related encephalopathy. The most frequent TRAEs were neutrophil count decrease (20.2%) and platelet count decreased (7.6%). Among the 238 patients with evaluable response, overall ORR was 32.4% and DCR was 82.4%. In the SCLC cohort (n= 73), ORR was 56.2%, with a higher rate in the subset of patients treated with 9 mg/kg dose rather than 6 mg/kg (70.4% vs 46.7%). Contrastingly, ORR in the other cancer types were lower: 22% in 41 patients with non-squamous NSCLC, 16% in 25 patients with squamous-NSCLC, 28% in 32 patients with mCRPC. Furthermore, preliminary data of efficacy were also reported in few patients with melanoma, HNSCC, HCC and cervical cancer^31^.

## B7-H4

B7-H4, also known as VTCN1, is a critical member of the B7 family and a transmembrane protein that negatively regulates T-cell function. It is frequently overexpressed in cancer cells and immunosuppressive tumor-associated macrophages (TAMs). B7-H4 plays a significant role in cancer progression, inflammation, autoimmune diseases, and organ transplantation^32^. It is highly expressed in various tumor types, including cholangiocarcinoma (CCA), breast, ovarian, and endometrial cancers. Due to its limited expression in normal tissues, B7-H4 is considered an attractive target for IDCs^33^.

Puxitatug samrotecan, also known as AZD8205, is an IDC consisting of a human anti-B7-H4 mAb conjugated through a cleavable linker to TOP1i, AZ’0133, with a DAR of 8. The first-in-human clinical trial, BLUESTAR (NCT05123482), is an ongoing phase I/IIa study evaluating AZD8205 monotherapy in pretreated patients with advanced or metastatic biliary tract, breast, ovarian, or endometrial cancers expressing B7-H4 (Table 1). Among the 47 patients enrolled in the different dose levels (0.8–3.2 mg/kg every three weeks), 91.5% experienced a any-grade TRAE and 55.3% a ≥ G3 TRAE, the most common of which was neutropenia (34%). Two patients had TRAEs that led to discontinuation, one G3 acute kidney injury and one G5 ILD. Responses were observed across a broad range of B7-H4 expression and at all dose levels, with an ORR of 19.5%. Phase II expansion cohorts are currently under investigation^34^.

HS-20089 is an investigational IDC comprising a humanized mAb linked to a topoisomerase I inhibitor via a protease-cleavable linker, with DAR of 6. HS-20089 is under evaluation in a phase I trial in patients with advanced solid tumors refractory to standard therapies (Table 1). Among the 52 patients evaluable in the dose escalation cohorts (0.7–7.2 mg/kg every three weeks), TRAEs occurred in 98.1%. The most common TRAEs occurring in more than 20% of the patients were leukopenia, neutropenia, nausea, anemia and thrombocytopenia. The MTD was defined as 5.8 mg/kg. At potential target therapeutic doses of 4.8 and 5.8 mg/kg, the ORR in TNBC were 33.3% and 27.3%, respectively^35^. Other trials evaluating HS-20089 alone or in combination with other compounds are ongoing, including a phase III trial in patients with platinum-resistant recurrent epithelial ovarian cancer, fallopian tube cancer, or primary peritoneal cancer (Table 2).

Felmetatug vedotin, also known as SGN-B7H4V, is an IDC comprising a humanized mAb targeting B7-H4, linked to MMAE via a protease-cleavable peptide linker. The phase I study SGNB7H4V-001 (NCT05194072) is evaluating felmetatug vedotin in refractory advanced solid tumors irrespective of B7-H4 expression (Table 1). Seventy-five patients received SGN-B7H4V at different doses and schedules. ≥ G3 TEAE were less frequent in 21-day cycles than in 28-day cycles regimen. Confirmed objective responses were reported in patients with breast (7/25), ovarian (2/15), endometrial (1/16), and biliary tract cancers (2/9). Dose expansion in selected tumors is planned^36^.

## CD56

CD56, also known as the neural cell adhesion molecule (NCAM), plays critical roles in development, nervous system differentiation, and immune surveillance^37^. It is primarily expressed in neuroendocrine cells, NK cells, and T-cell lineages. Aberrant expression of CD56 is observed in various hematological malignancies and solid tumors, most notably in SCLC^38^.

Lorvotuzumab mertansine, also known as IMGN901, is composed of a humanized anti-CD56 antibody linked to the tubulin-binding maytansinoid DM1 via a stable disulfide linker. IMGN901 was evaluated in a phase I clinical trial in 97 patients with relapsed or refractory SCLC, neuroendocrine pulmonary tumors, metastatic Merkel cell carcinoma (MCC), carcinoid tumors, or other CD56-positive solid tumors (Table 1). In the dose-escalation phase (4-94 mg/m² administered on 3 consecutive days every 21 days), MTD of 75 mg/m² was determined. The most common ≥ G3 TEAEs were hyponatremia and dyspnea (both 8.2%). One radiological complete response (CR), one clinical CR and one unconfirmed partial response (PR) were reported in MCC, while disease stability was reported in 25% of evaluable patients receiving doses of 60 mg/m² or higher^39^. Lorvotuzumab mertansine was subsequently evaluated in a phase I-II trial in patients with previously untreated SCLC, in combination with carboplatin-etoposide compared to carboplatin-etoposide alone. However, the combination did not improve efficacy, as the median mPFS was 6.2 months for the combination arm compared to 6.7 months for the chemotherapy arm (HR 0.93, 95%CI 0.58-1.51). Additionally, combination therapy led to increased toxicity, including a higher incidence of serious infections, such as pneumonia or sepsis, with some fatal cases (9 in the combination arm vs. 1 in the chemotherapy-only arm). Consequently, further development of lorvotuzumab mertansine was discontinued^40^.

## CD70

The immune checkpoint molecule CD70 and its receptor CD27, both members of the tumor necrosis factor (TNF) superfamily, are aberrantly expressed in a variety of hematological and solid malignancies. Dysregulation of the CD70-CD27 signaling axis within tumors and their microenvironment is associated with tumor progression and immunosuppression^41^. Aberrant CD70 expression was reported in several solid tumors, including RCC, NPC, glioblastoma, melanoma, lung carcinoma, cervical carcinoma, breast carcinoma, ovarian carcinoma, and mesothelioma, while virtually absent in prostate cancer and CRC. Few IDCs targeting CD70 were evaluated in the past years, but abandoned due to low response rates and toxicity concerns, while one (PRO-1160) has recently entered clinical investigation.

AMG-172 consists of a human IgG1 mAb conjugated to lysine residues via a non-cleavable linker (4-[N-maleimidomethyl] cyclohexane-1-carboxylate) and DM1, a semi-synthetic derivative of maytansine. AMG-172 was assessed in a phase I, first-in-human study (NCT01497821) in patients with relapsed/refractory clear cell renal cell carcinoma irrespective of CD70 expression (Table 1). In the dose-exploration phase (0.15-2.4 mg/kg administered every two weeks), the MTD was identified as 1.6 mg/kg, with thrombocytopenia being the most common dose-limiting toxicity. All the 37 patients experienced at least one TEAE, with the most frequently reported being thrombocytopenia, nausea, decreased appetite, vomiting, fatigue and anemia. The ORR was only 5.4%^42^.

Vorsetuzumab mafodotin (SNG-75) is an IDC directed against CD70 antigen conjugated to monomethyl auristatin F (MMAF) which was tested in a phase I trial (NCT01015911) in relapsed/refractory non-Hodgkin lymphoma and metastatic clear cell renal cell carcinoma. Vorsetuzumab mafodotin was administered on 3 weeks cycles at dose escalation of 0.3–4.5 mg/kg (47 patients) or on weekly regimens at dose of 0.3 or 0.6 mg/kg (11 patients) in 58 patients (19 non-Hodgkin lymphoma and 39 clear cell renal cell carcinoma). At every-three-weeks regimen, adverse events of grade 3 or higher were reported in 70% of the patients, with 19% of thrombocytopenia and 23% of eye disorder. At weekly dosing, ≥ G3 adverse events were reported in 55% of the patients with 2 cases (18%) of idiopathic thrombocytopenic purpura. In term of ORR results were poor, with only one CR (7%) in non-Hodgkin lymphoma and 2 PR (6%) in clear cell renal cell carcinoma receiving SNG-75 within the every-three-weeks regimen. No responses were reported with weekly doses^43^.

BMS-936561 is composed of an anti CD70 mAb conjugated to MED-2460, a prodrug of a cytotoxic DNA minor groove-binding alkylating agent. Twenty-six patients with non-Hodgkin lymphoma or clear cell renal cell carcinoma received BMS-936561 in a phase I trial (NCT00944905) in dose escalation phase (0.5-15 mg/kg). Adverse event of grade 3 or higher were reported in 9 patients (34.6%) and included pleural effusion (11.5%) and thrombocytopenia (7.7%). No objective responses were observed^44^.

## Discussion

Currently, ADCs represent one of the most promising therapeutic strategies in cancer treatment, offering high tumor specificity and minimizing systemic toxicity. Most of approved or in-development ADCs target tumor-associated antigens, and their combination with immunotherapy is being actively explored across various cancer types. This approach leverages the synergistic potential of ADCs and immunotherapy but also increases the risk of specific toxicities of concern. Therefore, optimizing ADC-based combination strategies is essential to maximize antitumor efficacy while minimizing adverse effects^45–47^. IDCs are a novel class of ADCs that combines the effect of ICIs in blocking the T-Cell inhibitory signal and the immunomodulating proprieties of the cytotoxic payload remodeling TME, opening a new landscape of possibility in immuno-oncology^8^. Unlike traditional ADC targets which exploit overexpressed tumor antigens, immune-checkpoints are directly implicated in tumor immune evasion. By targeting these molecules, IDCs not only deliver cytotoxic payloads to tumor cells and TME but may also contribute to reverse immune suppression, potentially leading to synergistic antitumor effects.

Recently, several IDCs have been developed and tested in preclinical settings, though only a few have progressed into clinical trials. Among these, IDCs targeting PD-L1, B7-H3 and B7-H4 are the most studied today, although other immune-checkpoint targets including CD56, CD70 and CD73 are under evaluation as well. Although further elucidations of immunosuppressive effects on TME of B7-H family members are needed to understand their receptor-ligand interaction and their synergism with other co-stimulatory molecules including B7-H2 and B7-H6, PD-L1, B7-H3 and B7-H4 demonstrated to be promising target in the modulation of TME^48^.

Although ICIs targeting PD1-PDL1 axis have demonstrated great efficacy among different tumor types and are widely used in daily clinical practice, IDCs targeting PD-L1 are still in an early stage of development, with SGN-PDL1V and HLX43 being the only two tested in humans. Conversely, B7-H3 seems to be a promising novel target for IDCs due to its role in multiple pathways, including immunomodulation and pathologic angiogenesis. Several classes of anti-B7-H3 drugs are currently in development, including mAbs, radioimmunotherapy and IDCs^16^. Different anti-B7-H3 IDCs have been tested in humans with promising results, including Ifinatamab deruxtecan, YL201, HS-20093, Mirzotamab clezutoclax, MHB088C and DB-1311/BNT324, some of which are currently under investigation in phase III clinical trials. Moreover, other IDCs targeting B7-H4 such as Puxitatug samrotecan, HS-20089 and felmetatug vedotin, are in an early examination phase. New proteins with immunomodulatory properties in TME are under evaluation as targets for IDCs, making CD70, CD56 and CD73 potentially viable targets of IDCs. However, IDCs targeting CD70 and CD56 explored in the early 2010s, including lorvotuzumab mertansine, AMG-172, vorsetuzumab mafodotin (SGN-75) and BMS-936561, showed limited efficacy and were associated with high rates of adverse events. Few other IDCs targeting CD70 and CD56 with different payloads are currently under evaluation to further elucidate the role of CD70 and CD56 as a potential target for remodeling the TME. CD73, which contributes to immune suppression in the tumor microenvironment, is currently being targeted by a few inhibitors in clinical development, including anti-CD73 monoclonal antibodies and IDCs^49,50^.

While IDCs offer targeted tumor killing, safety remains a primary concern, particularly due to potential on-target/off-tumor toxicity. Many immune-checkpoint molecules, such as PD-1, CD70, and CD73, are expressed on normal immune cells, raising the risk of immune-related adverse events (irAEs), hematologic toxicities, and organ-specific toxicities. Currently, clinical trials exploring IDCs mainly focus on determining MTD and safety profile rather than exploring efficacy. In general, safety profile is acceptable with toxicities mainly related to the payload rather than to the immune mechanisms. The most commonly reported grade ≥3 adverse events include neutropenia, anemia, and thrombocytopenia, reflecting the systemic cytotoxic effects of the conjugated payloads. For example, a decrease in neutrophil count was frequently observed in patients treated with YL201, MHB088C and AZD8205, with rates reaching up to 49% in patients treated with IMGN901. Anemia also emerged as a recurrent toxicity, with incidences up to 25% in YL201-treated cohorts. Other frequently reported hematologic events included leukopenia and decreased platelet count. Severe non-hematologic toxicities such as dyspnea and hypotension were less frequently, as seen in some SGN-B7H4V-treated patients. Notably, the overall rate of TRAE) exceeded 50% in several trials, peaking at 91.7% in the DB-1311 study. These findings highlight the importance of careful patient selection and proactive toxicity monitoring during IDC therapy, aiming to optimize clinical benefit while minimizing treatment-related risks.

Preliminary results of ongoing clinical trials showed that IDCs may be particularly effective in tumors with "cold" TME (e.g. SCLC) where the activity of ICIs is not enough powered to ensure a potent and durable antitumor effect^51^. "Cold" tumors, characterized by low immune infiltration and resistance to ICIs, represent an area of high unmet clinical need where IDCs may offer unique therapeutic advantages. Among these, SCLC and mCRPC stand out, with several IDCs—particularly those targeting B7-H3—demonstrating promising response rates despite prior treatment failure. In SCLC, agents such as DS-7300a and YL201 have shown objective responses exceeding 50% in some cohorts. Similarly, IDCs have reported encouraging activity in mCRPC, a tumor type traditionally unresponsive to immunotherapy. Other "cold" or "immune-desert" tumors such as heavily pretreated TNBC, ovarian, endometrial, and sarcomas have also exhibited signs of sensitivity to IDC-based strategies. These findings suggest that IDCs may overcome the immunological silence of "cold" tumors by releasing their payload directly within the TME. This reprogramming through various immunomodulatory mechanisms may convert the TME into a "hot" state, enhancing the effectiveness of both immune-checkpoint blockade and cytotoxic payload-induced cell killing^8^. Moreover, Ifinatamab deruxtecan and YL201 showed response even in CNS localization, suggesting that IDCs may provide intracranial efficacy as well.

Unlike HER2-targeting ADCs, where clear biomarker-driven selection strategies exist, the variability of checkpoint expression and TME interactions complicates patients’ stratification. The efficacy of this novel class of IDCs may be independent of both target expression levels on tumor tissue and circulating soluble target in blood, regardless of IDC type, as suggested for SGNPDL1V, Ifinatamab deruxtecan, YL201, HS-20093 and puxitatug samrotecan. These findings highlight the potential application of IDCs across different tumor types with variable target expression levels. Furthermore, since IDCs demonstrated activity regardless of target expression levels, heterogeneity of immune-checkpoint expression, both intra-patient and inter-patients, do not seem to significantly impact their efficacy. Due to the limited evidence currently available on IDCs mechanisms of action, it is difficult to definitively assess why therapeutic efficacy is not influenced by target expression. Nonetheless, some hypotheses can be made by drawing parallelisms with conventional ADCs and ICIs. First, the bystander effect observed with classical ADCs is likely to occur with IDCs, potentially enabling cytotoxicity in target-negative tumor cells. Moreover, in contrast to classical ADCs, IDCs can target overexpressed immune-checkpoint on cells within the TME. This facilitates the delivery of cytotoxic payloads, reactivates immune responses, and induces antitumor effects by promoting bystander killing, ultimately contributing to tumor cell death. Evaluation of immune-checkpoints expression on both tumor and TME cells, mimicking Combined Positive Score (CPS), may serve as a predictive biomarker for IDCs efficacy. However, the evidence is still too scarce to draw solid conclusions and future trials stratifying patients according to target expression levels are warranted.

Given the challenges posed by tumor heterogeneity and the emergence of resistance mechanisms, combination strategies could be explored to enhance the therapeutic efficacy of IDCs. Chemotherapy or radiotherapy may also serve as effective partners of IDCs, as these modalities can upregulate target antigen expression and increase tumor permeability, thereby enhancing IDC delivery and uptake. Moreover, combining IDCs with targeted therapies—such as AXL, VEGF, or CD47 inhibitors—could help to remodel the tumor microenvironment, reduce stromal barriers, and improve intratumoral drug penetration. Finally, next-generation designs such as bispecific IDCs or dual-payload formats offer the potential to overcome antigen heterogeneity and mitigate resistance by simultaneously targeting multiple tumor pathways or delivering different cytotoxic mechanisms. These innovative strategies are central to expanding the clinical impact of IDCs across a wider spectrum of solid tumors.

There is significant industrial interest in developing IDCs class, which have the potential of improving benefit especially in poor-outcome patients, such as those with immunologically "cold" tumors or those who progressed on ICIs. If the role of IDCs will be confirmed in larger phase III clinical trials, some of which are currently ongoing, this novel class of therapeutic agents may revolutionize oncology treatment, especially in those cancers with poor prognosis, such as SCLC, sarcomas, NPCs and HNSCC, leading to an exceptional improvement in patient outcomes in terms of both survival and quality of life. Moreover, several trials are currently investigating IDCs in combination with other standard treatments such as chemotherapy, ICI or antiangiogenics drugs (including NCT06848699, NCT06362252, NCT04165070, NCT06863272, NCT06394414, NCT06332170, NCT06699576, NCT06825624, NCT06336707). Such combinations may further enhance treatment efficacy and improve patients’ prognosis. Lastly, a few novel IDCs, including those based on bispecific mAbs or incorporating dual payloads are also under evaluation. However, their safety profile requires thorough investigation due to the potential for increased toxicities.

## Conclusions

IDCs hold potential as a novel class of cancer therapeutics, combining immune modulation with potent cytotoxicity. Nevertheless, toxicity concerns, biomarker selection and tumor resistance mechanisms remain key hurdles. Ongoing clinical trials and combination strategies will determine the long-term success of IDCs in cancer treatment. Moving forward, rational drug design, innovative targeting approaches and patient-specific therapeutic strategies will be essential in establishing IDCs as a cornerstone of next-generation oncology treatment.

## Supplementary information

Suplementary material

## Data Availability

No datasets were generated or analysed during the current study.
