# Supplementary material for: Immune-checkpoint targeting drug conjugates: a novel class of promising therapeutic agents for cancer treatment
Source: NPJ Precis Oncol. 2025 Jul 2;9:219. doi: 10.1038/s41698-025-01011-7 (PMC12223136; doi:10.1038/s41698-025-01011-7)
Supplement: Supplementary file 1 — Suplementary material [file 41698_2025_1011_MOESM1_ESM.docx]

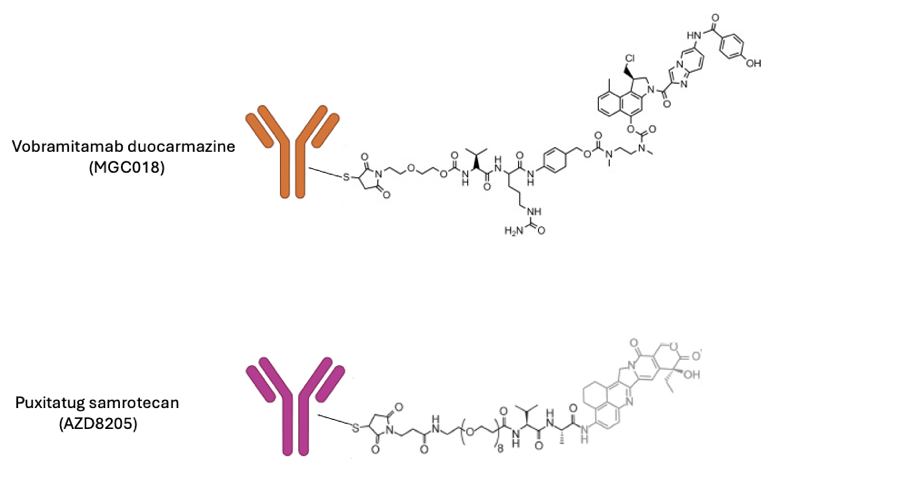

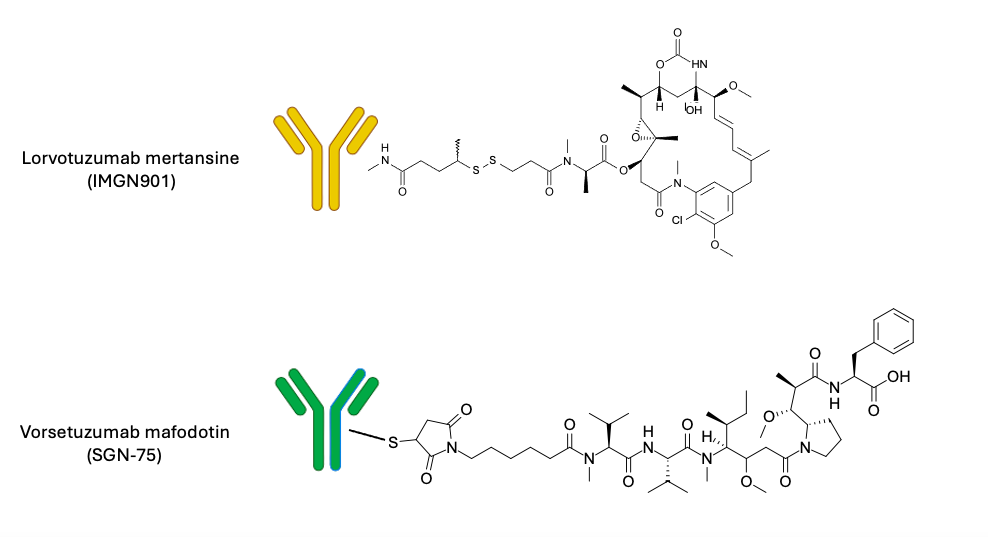

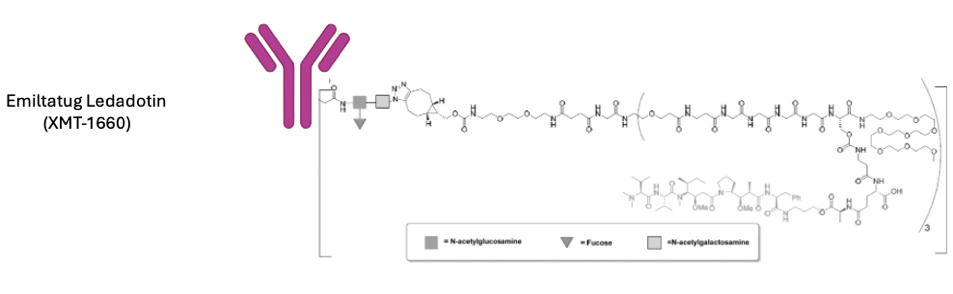

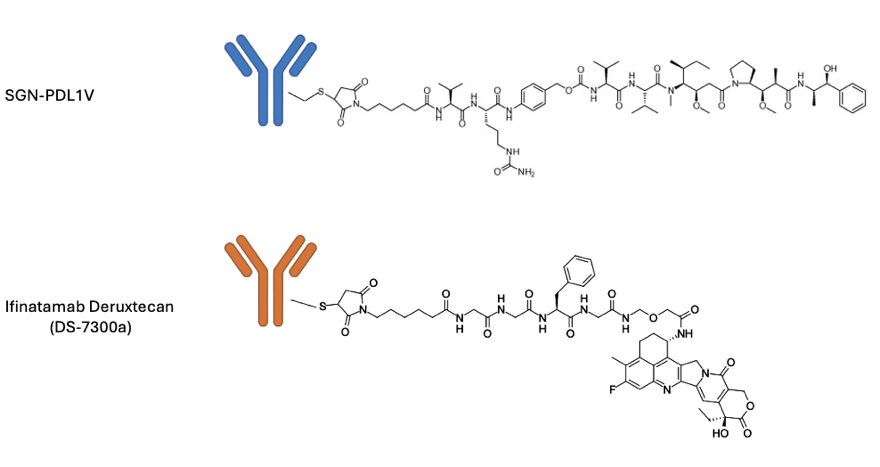
**Supplementary figure 1: Available molecular structures of IDCs**


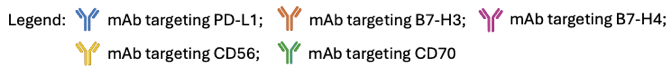


*Abbreviations: mAb = monoclonal antibody*
